# Supplementary material for: Graph transformation for enzymatic mechanisms
Source: Bioinformatics. 2021 Jul 12;37(Suppl 1):i392–400. doi: 10.1093/bioinformatics/btab296 (PMC8686676; doi:10.1093/bioinformatics/btab296)
Supplement: btab296_Supplementary_Data [file btab296_Supplementary_Data.zip › btab296-suppl_data/amino_acid_tautomers.pdf]

# Summary

January 27, 2021

# Contents


0.1 Named Graphs . . . 2
- 0.1.1 Protonated Arginine . . . 2
- 0.1.2 Arginine . . . 2
- 0.1.3 Asparagine . . . 2
- 0.1.4 Deprotonated Aspartate . . . 2
- 0.1.5 Aspartate . . . 3
- 0.1.6 Deprotonated Cysteine . . . 3
- 0.1.7 Cysteine . . . 3
- 0.1.8 Glutamine . . . 3
- 0.1.9 Glutamate . . . 4
- 0.1.10 Deprotonated Glutamate . . . 4
- 0.1.11 Histidine-delta . . . 4
- 0.1.12 Protonated Histidine-delta . . . 4
- 0.1.13 Histidine-epsilon . . . 5
- 0.1.14 Protonated Histidine-epsilon . . . 5
- 0.1.15 Isoleucine . . . 5
- 0.1.16 Leucine . . . 6
- 0.1.17 Lysine . . . 6
- 0.1.18 Protonated Lysine . . . 6
- 0.1.19 Methionine . . . 6
- 0.1.20 Phenylalanine . . . 7
- 0.1.21 Serine . . . 7
- 0.1.22 Threonine . . . 7
- 0.1.23 Tryptophan . . . 7
- 0.1.24 Tyrosine . . . 8
- 0.1.25 Deprotonated Tyrosine . . . 8
- 0.1.26 Valine . . . 8

## 0.1 Named Graphs

### 0.1.1 Protonated Arginine

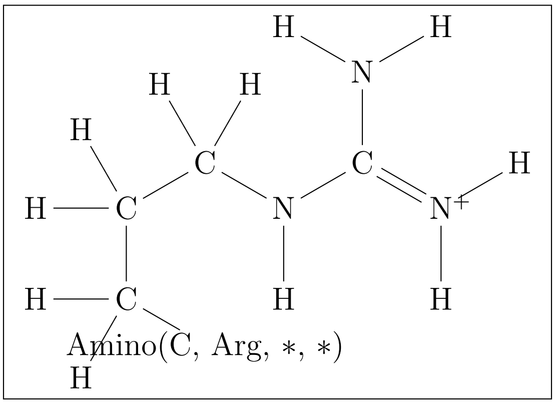


File: out/000_g_0_10300000

### 0.1.2 Arginine

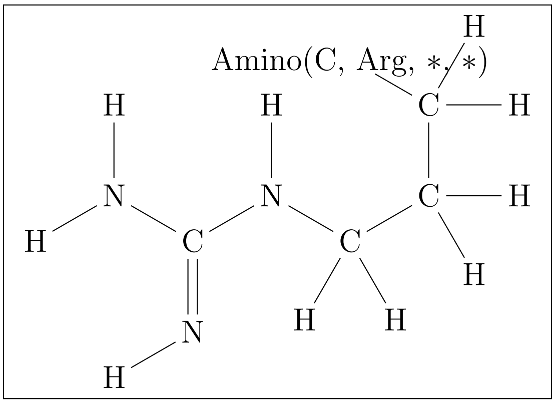


File: out/001_g_1_10300000

### 0.1.3 Asparagine

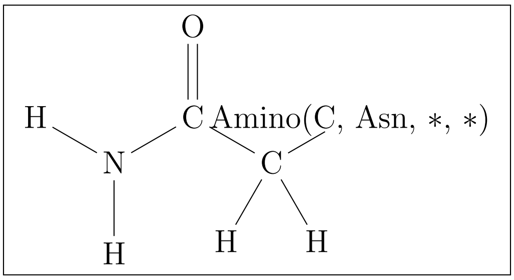


File: out/002_g_2_10300000

### 0.1.4 Deprotonated Aspartate

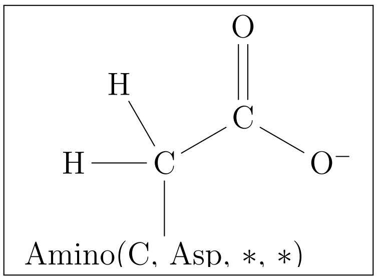

File: out/003_g_3_10300000

### 0.1.5 Aspartate

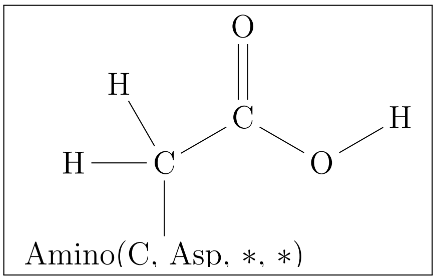


File: out/004_g_4_10300000

### 0.1.6 Deprotonated Cysteine

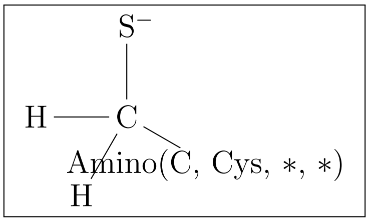


File: out/005_g_5_10300000

### 0.1.7 Cysteine

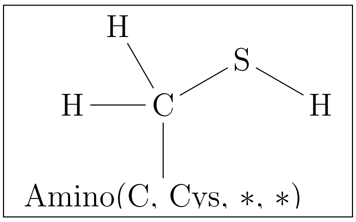


File: out/006_g_6_10300000

### 0.1.8 Glutamine

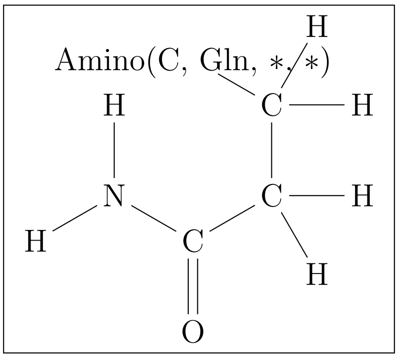


File: out/007_g_7_10300000

### 0.1.9 Glutamate

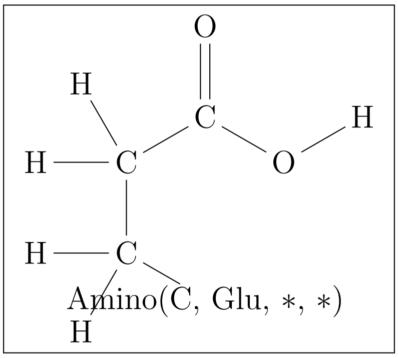


File: out/008_g_8_10300000

### 0.1.10 Deprotonated Glutamate

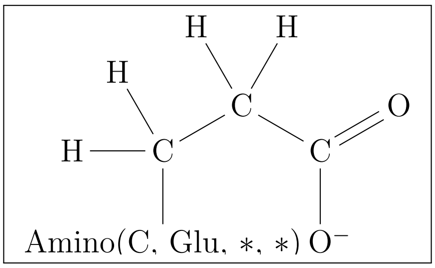


File: out/009_g_9_10300000

### 0.1.11 Histidine-delta

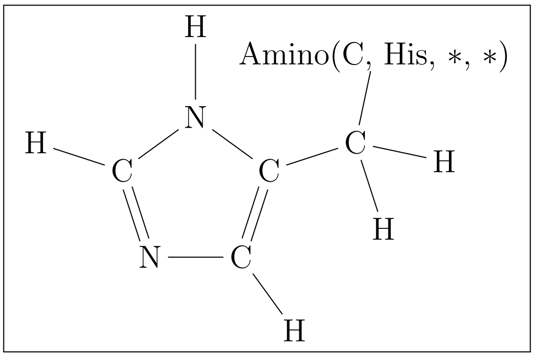


File: out/010_g_10_10300000

### 0.1.12 Protonated Histidine-delta

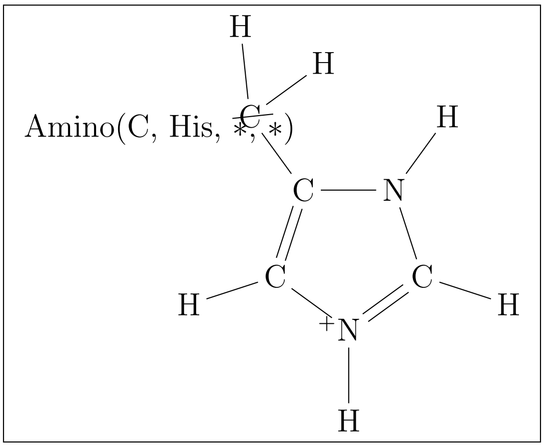


File: out/011_g_11_10300000

### 0.1.13 Histidine-epsilon

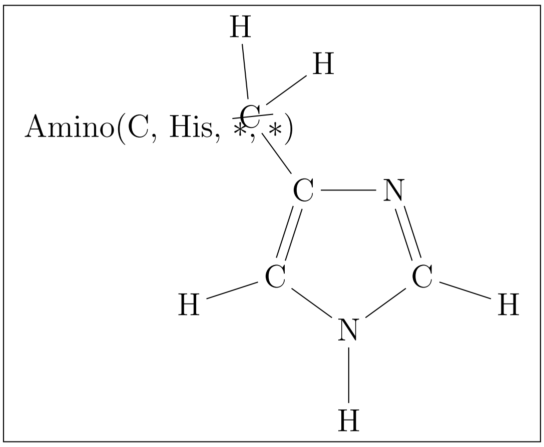


File: out/012_g_12_10300000

### 0.1.14 Protonated Histidine-epsilon

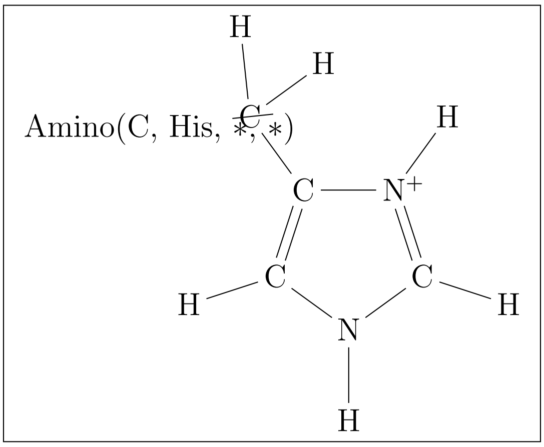


File: out/013_g_13_10300000

### 0.1.15 Isoleucine

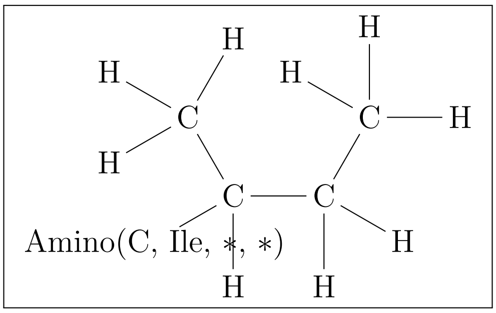


File: out/014_g_14_10300000

### 0.1.16 Leucine

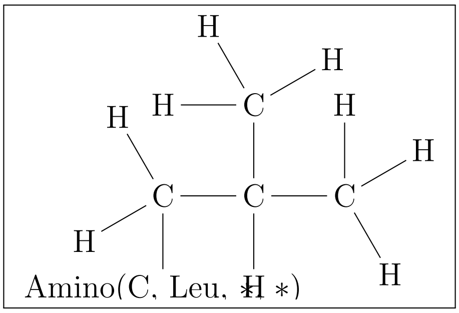


File: `out/015_g_15_10300000`

### 0.1.17 Lysine

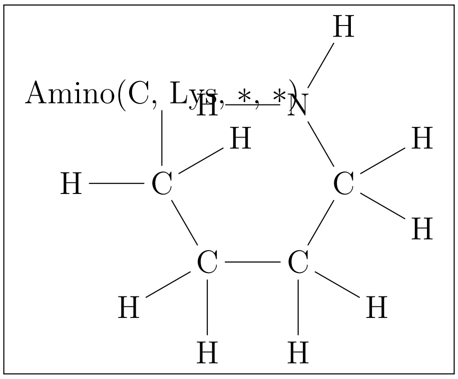


File: `out/016_g_16_10300000`

### 0.1.18 Protonated Lysine

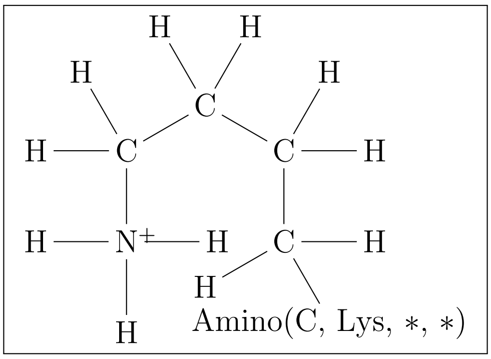


File: `out/017_g_17_10300000`

### 0.1.19 Methionine

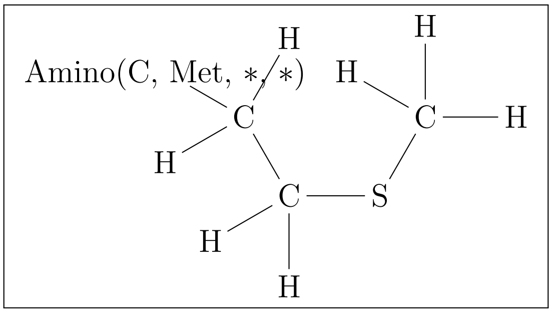


File: `out/018_g_18_10300000`

### 0.1.20 Phenylalanine

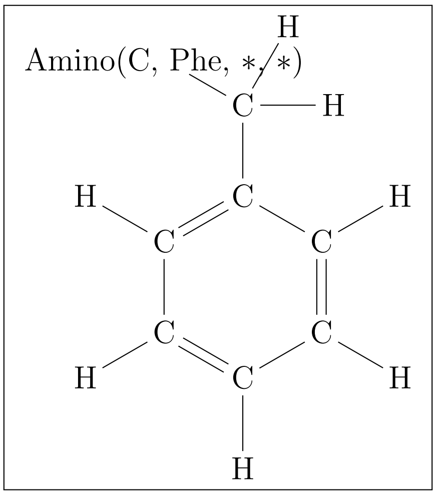


File: out/019_g_19_10300000

### 0.1.21 Serine

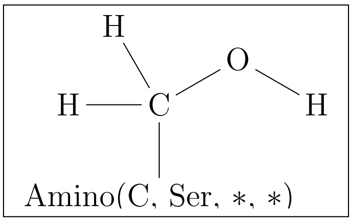


File: out/020_g_20_10300000

### 0.1.22 Threonine

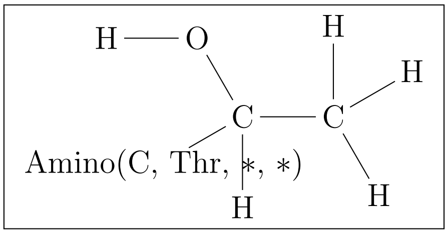


File: out/021_g_21_10300000

### 0.1.23 Tryptophan

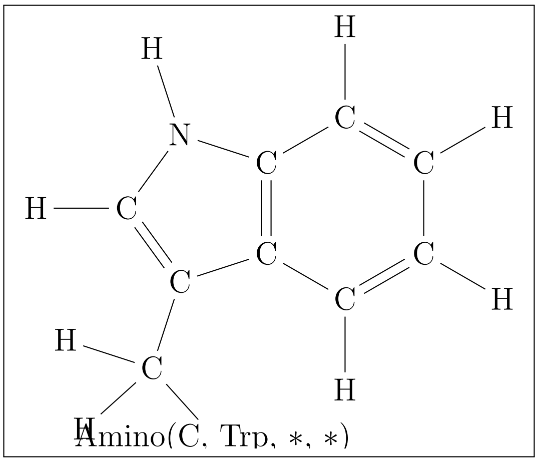


File: out/022_g_22_10300000

### 0.1.24 Tyrosine

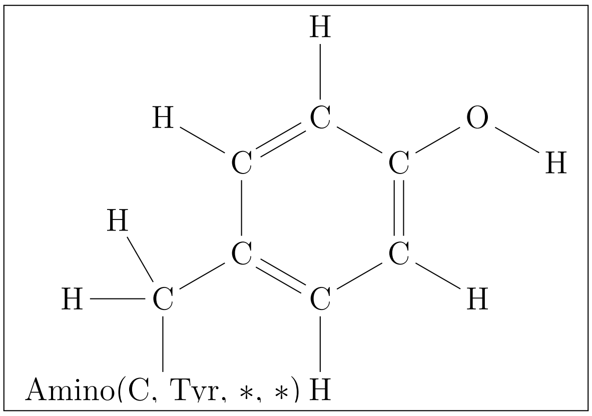


File: `out/023_g_23_10300000`

### 0.1.25 Deprotonated Tyrosine

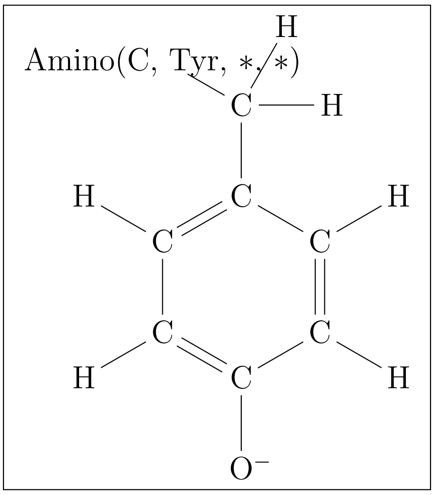


File: `out/024_g_24_10300000`

### 0.1.26 Valine

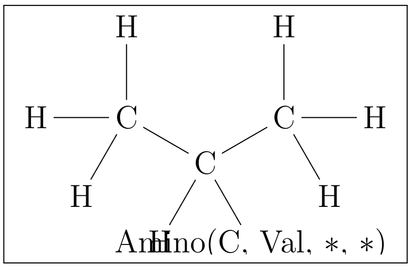


File: `out/025_g_25_10300000`
