# Supplementary material for: Graph transformation for enzymatic mechanisms
Source: Bioinformatics. 2021 Jul 12;37(Suppl 1):i392–400. doi: 10.1093/bioinformatics/btab296 (PMC8686676; doi:10.1093/bioinformatics/btab296)
Supplement: btab296_Supplementary_Data [file btab296_Supplementary_Data.zip › btab296-suppl_data/functional_patterns.pdf]

Summary

January 27, 2021

Contents

0.1 Named Graphs . . . 5
- 0.1.1 proton . . . 5
- 0.1.2 water . . . 5
- 0.1.3 hydronium . . . 5
- 0.1.4 hydroxide . . . 5
- 0.1.5 carbon dioxide . . . 5
- 0.1.6 oxygen molecule . . . 5
- 0.1.7 peroxide . . . 5
- 0.1.8 peroxide_base_0 . . . 5
- 0.1.9 benzene . . . 6
- 0.1.10 cyclopentane . . . 6
- 0.1.11 cyclopentane_base_0 . . . 6
- 0.1.12 cyclopentene . . . 6
- 0.1.13 cyclohexane . . . 6
- 0.1.14 cyclohexane_base_0 . . . 7
- 0.1.15 cyclohexene . . . 7
- 0.1.16 1,5-cyclodecadiene . . . 7
- 0.1.17 1,5-cycloundecadiene_base_0 . . . 7
- 0.1.18 haloalkane . . . 7
- 0.1.19 alcohol . . . 8
- 0.1.20 alcohol_base_0 . . . 8
- 0.1.21 alkanediol . . . 8
- 0.1.22 alkanediol_base_0 . . . 8
- 0.1.23 alkanediol_base_1 . . . 8
- 0.1.24 aldehyde . . . 8
- 0.1.25 ketone . . . 8
- 0.1.26 carboxylic acid . . . 9
- 0.1.27 carboxylic acid_base_0 . . . 9
- 0.1.28 carbamic acid . . . 9
- 0.1.29 carbamic acid_base_0 . . . 9
- 0.1.30 carbamoyl . . . 9
- 0.1.31 acid anhydride . . . 10
- 0.1.32 ester . . . 10
- 0.1.33 methoxyol . . . 10
- 0.1.34 methoxyol_base_0 . . . 10
- 0.1.35 methoxydiol_base_0 . . . 10
- 0.1.36 ether . . . 11
- 0.1.37 ether_acid_0 . . . 11
- 0.1.38 epoxide . . . 11
- 0.1.39 amine . . . 11
- 0.1.40 amine_acid_0 . . . 11

0.1.41 amine_base_0 11
0.1.42 amide 12
0.1.43 amide_acid_0 12
0.1.44 nitrous acid 12
0.1.45 nitrous acid_base_0 12
0.1.46 nitrile 12
0.1.47 nitrile_base_0 12
0.1.48 nitroso 12
0.1.49 imine 13
0.1.50 imine_acid_0 13
0.1.51 thiol 13
0.1.52 thiol_base_0 13
0.1.53 selenol 13
0.1.54 selenol_base_0 13
0.1.55 sulfide 13
0.1.56 sulfide_acid_0 14
0.1.57 disulfide 14
0.1.58 disulfide_base_0 14
0.1.59 sulfanyl alkanone 14
0.1.60 sulfanyl alkanol 14
0.1.61 sulfanyl alkanol_base_0 14
0.1.62 sulfoxide 15
0.1.63 sulfonium 15
0.1.64 sulfurous acid_base_0 15
0.1.65 sulfurous acid_base_1 15
0.1.66 sulfuric acid_base_0 15
0.1.67 sulfuric acid_base_1 16
0.1.68 sulfonic acid_base_0 16
0.1.69 sulfonate ester 16
0.1.70 thial 16
0.1.71 phosphine 16
0.1.72 phosphoric acid_base_0 17
0.1.73 phosphoric acid_base_1 17
0.1.74 phosphoric acid_base_2 17
0.1.75 metaphosphate 17
0.1.76 phosphoester 17
0.1.77 phosphoester_base_0 18
0.1.78 phosphoester_base_1 18
0.1.79 thiophosphoester_base_1 18
0.1.80 pentahydroxyphosphorane_base_1 18
0.1.81 pentahydroxyphosphorane_base_2 18
0.1.82 tetrahydroxyphosphorane_base_2 19
0.1.83 hydroxyoxophosphoniumolate_base_0 19
0.1.84 vanadate 19
0.1.85 vanadate_acid_0 19
0.1.86 vanadate_acid_1 19
0.1.87 peroxovanadate 20
0.1.88 peroxovanadate_base_0 20
0.1.89 tetrahydrofuran 20
0.1.90 tetrahydrofuran_acid_0 20

0.1.91 oxacyclopentene 20
0.1.92 butyrolactone 21
0.1.93 dioxolane 21
0.1.94 dioxolane-2-ol 21
0.1.95 dioxolane-2-ol_acid_0 21
0.1.96 tetrahydropyran 21
0.1.97 tetrahydropyran_base_0 22
0.1.98 tetrahydropyran_base_1 22
0.1.99 dihydropyran 22
0.1.100 valerolactone 22
0.1.101 tetrahydropyran-3-one 22
0.1.102 4-oxotetrahydropyran 23
0.1.103 azetidinol 23
0.1.104 azetidinol_base_1 23
0.1.105 azacyclobutanone 23
0.1.106 pyrrolidine 23
0.1.107 pyrrole 24
0.1.108 imidazole 24
0.1.109 imidazole_acid_0 24
0.1.110 imidazole_base_0 24
0.1.111 monophosphoimidazole_base_1 24
0.1.112 imidazoline 25
0.1.113 imidazoline_acid_0 25
0.1.114 imidazoline_base_0 25
0.1.115 imidazol-2-ine 25
0.1.116 imidazol-2-ine_base_0 25
0.1.117 piperidine 26
0.1.118 piperideine 26
0.1.119 pyridine 26
0.1.120 pyridine_acid_0 26
0.1.121 1,2-dihydropyridine_acid_0 27
0.1.122 3,4-dihydropyridine 27
0.1.123 1,4-dihydropyridine 27
0.1.124 pyrimidine 27
0.1.125 pyrimidine_acid_0 28
0.1.126 2-alkylpyrimidine 28
0.1.127 dihydropyrimidine 28
0.1.128 dihydropyrimidine_acid_0 28
0.1.129 3,4,5,6-tetrahydropyrimidine 29
0.1.130 3,4,5,6-tetrahydropyrimidine_base_0 29
0.1.131 4-pyrimidinone 29
0.1.132 hydroxy-4-pyrimidinone 29
0.1.133 hydroxy-4-pyrimidinone_base_0 30
0.1.134 uracil 30
0.1.135 uracil_base_0 30
0.1.136 pyrazine 30
0.1.137 1,2-dihydropyrazine 30
0.1.138 1,2-dihydropyrazine_acid_0 31
0.1.139 2,3-dihydropyrazine 31
0.1.140 2,3-dihydropyrazine_acid_0 31

0.1.141 1,4-dihydropyrazine 31
0.1.142 tetrahydropyrazine 32
0.1.143 thiazole_acid_0 32
0.1.144 thiazolidine 32
0.1.145 thiazolidine_base_0 32
0.1.146 2,4-cyclohexadienone 32
0.1.147 2,5-cyclohexadienone 33
0.1.148 2,5-cyclohexadienone_acid_0 33
0.1.149 halobenzene 33
0.1.150 toluene 33
0.1.151 ortho-xylene 34
0.1.152 phenol 34
0.1.153 phenol_base_0 34
0.1.154 benzaldehyde 34
0.1.155 benzyl alcohol 35
0.1.156 benzyl alcohol_base_0 35
0.1.157 anisole 35

0.1 Named Graphs

0.1.1 proton

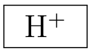

File: out/000_g_0_10300000

0.1.2 water

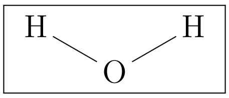

File: out/001_g_1_10300000

0.1.3 hydronium

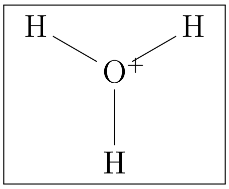

File: out/002_g_2_10300000

0.1.4 hydroxide

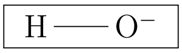

File: out/003_g_3_10300000

0.1.5 carbon dioxide

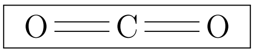

File: out/004_g_4_10300000

0.1.6 oxygen molecule

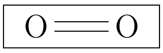

File: out/005_g_5_10300000

0.1.7 peroxide

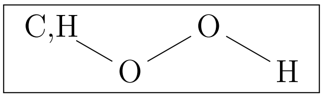

File: out/006_g_6_10300000

0.1.8 peroxide_base_0

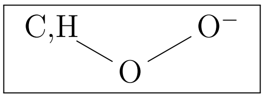

File: out/007_g_7_10300000

0.1.9 benzene

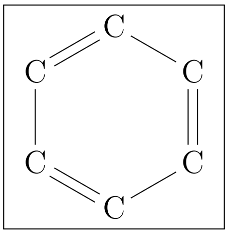

File: out/008_g_8_10300000

0.1.10 cyclopentane

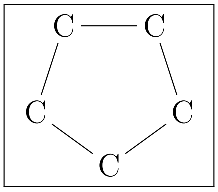

File: out/009_g_9_10300000

0.1.11 cyclopentane_base_0

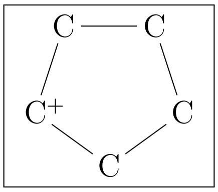

File: out/010_g_10_10300000

0.1.12 cyclopentene

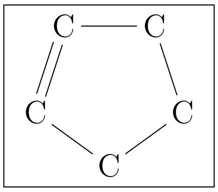

File: out/011_g_11_10300000

0.1.13 cyclohexane

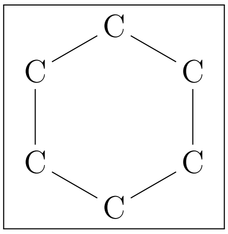

File: out/012_g_12_10300000

0.1.14 `cyclohexane_base_0`

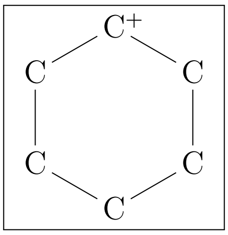

File: `out/013_g_13_10300000`

0.1.15 `cyclohexene`

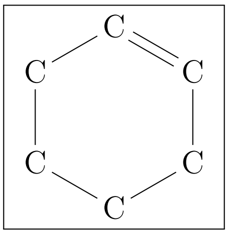

File: `out/014_g_14_10300000`

0.1.16 `1,5-cyclodecadiene`

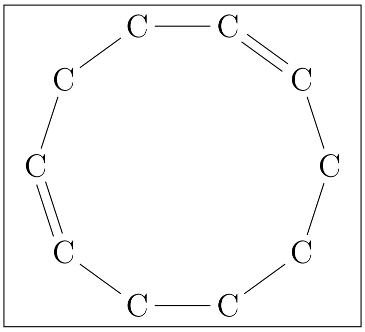

File: `out/015_g_15_10300000`

0.1.17 `1,5-cycloundecadiene_base_0`

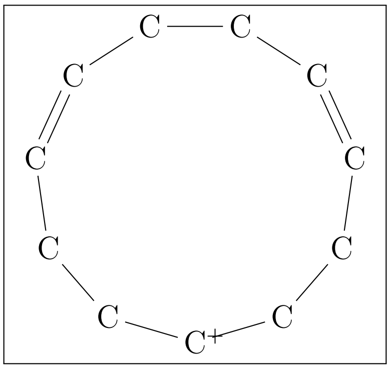

File: `out/016_g_16_10300000`

0.1.18 `haloalkane`

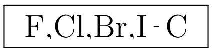

File: `out/017_g_17_10300000`

0.1.19 alcohol

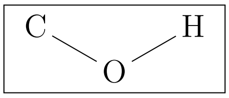

File: out/018_g_18_10300000

0.1.20 alcohol_base_0

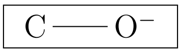

File: out/019_g_19_10300000

0.1.21 alkanediol

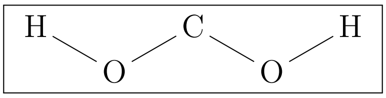

File: out/020_g_20_10300000

0.1.22 alkanediol_base_0

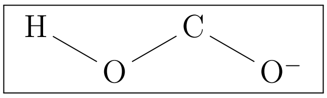

File: out/021_g_21_10300000

0.1.23 alkanediol_base_1

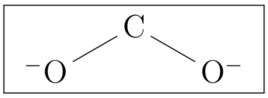

File: out/022_g_22_10300000

0.1.24 aldehyde

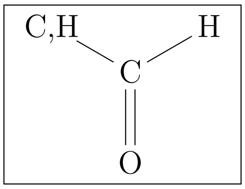

File: out/023_g_23_10300000

0.1.25 ketone

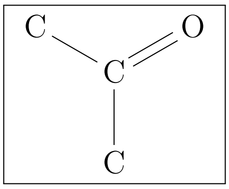

File: out/024_g_24_10300000

0.1.26 carboxylic acid

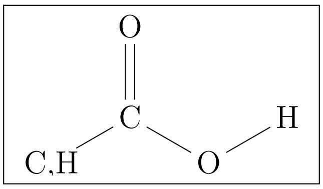

File: out/025_g_25_10300000

0.1.27 carboxylic acid_base_0

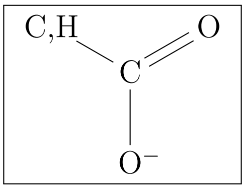

File: out/026_g_26_10300000

0.1.28 carbamic acid

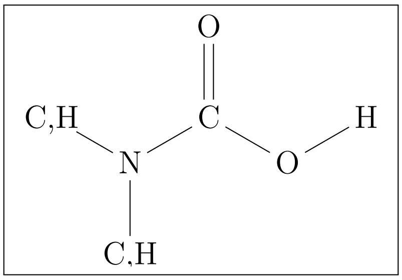

File: out/027_g_27_10300000

0.1.29 carbamic acid_base_0

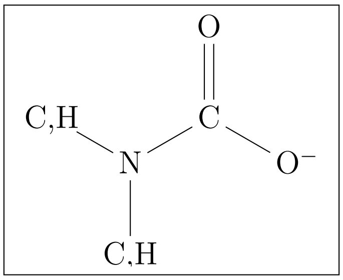

File: out/028_g_28_10300000

0.1.30 carbamoyl

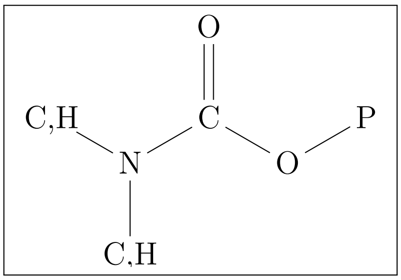

File: out/029_g_29_10300000

0.1.31 acid anhydride

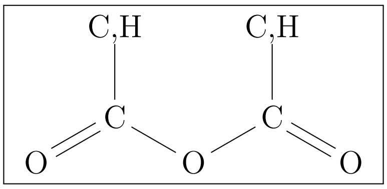

File: out/030_g_30_10300000

0.1.32 ester

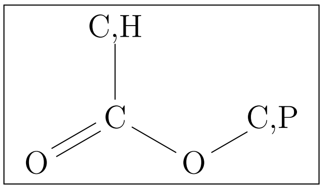

File: out/031_g_31_10300000

0.1.33 methoxyol

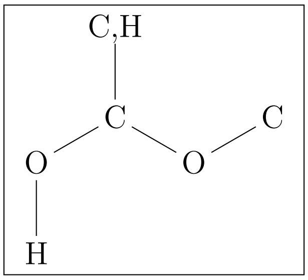

File: out/032_g_32_10300000

0.1.34 methoxyol_base_0

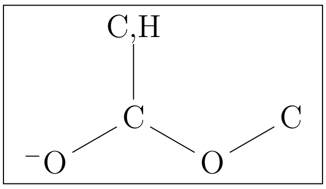

File: out/033_g_33_10300000

0.1.35 methoxydiol_base_0

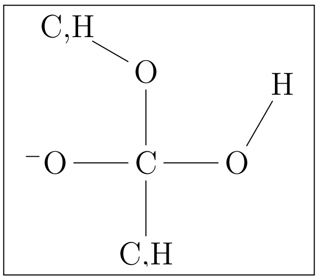

File: out/034_g_34_10300000

0.1.36 ether

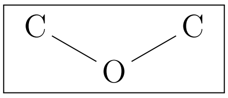

File: out/035_g_35_10300000

0.1.37 ether_acid_0

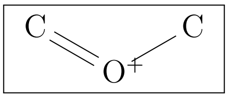

File: out/036_g_36_10300000

0.1.38 epoxide

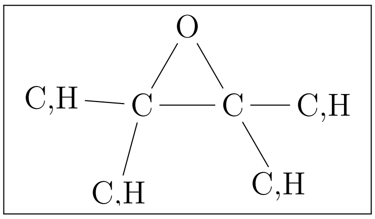

File: out/037_g_37_10300000

0.1.39 amine

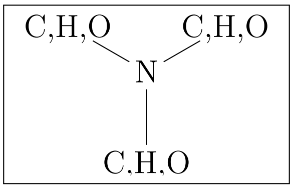

File: out/038_g_38_10300000

0.1.40 amine_acid_0

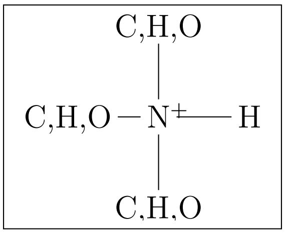

File: out/039_g_39_10300000

0.1.41 amine_base_0

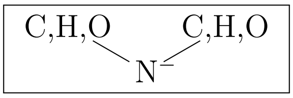

File: out/040_g_40_10300000

0.1.42 amide

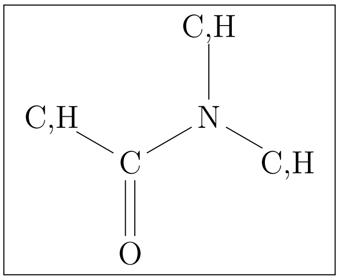

File: out/041_g_41_10300000

0.1.43 amide_acid_0

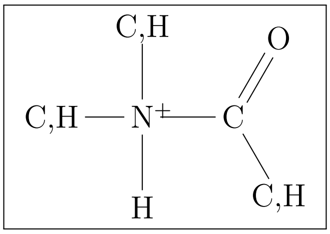

File: out/042_g_42_10300000

0.1.44 nitrous acid

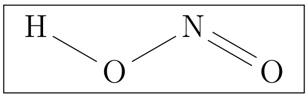

File: out/043_g_43_10300000

0.1.45 nitrous acid_base_0

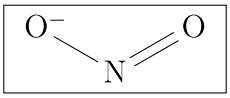

File: out/044_g_44_10300000

0.1.46 nitrile

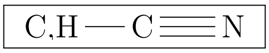

File: out/045_g_45_10300000

0.1.47 nitrile_base_0

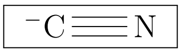

File: out/046_g_46_10300000

0.1.48 nitroso

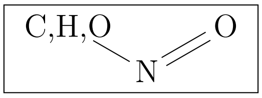

File: out/047_g_47_10300000

0.1.49 imine

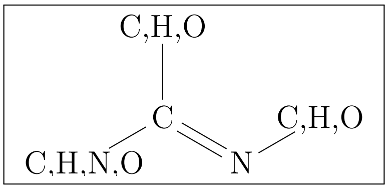

File: out/048_g_48_10300000

0.1.50 imine_acid_0

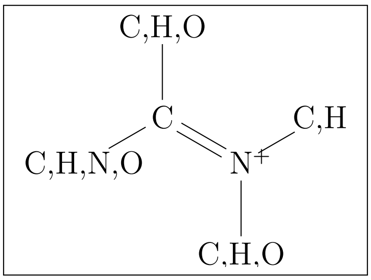

File: out/049_g_49_10300000

0.1.51 thiol

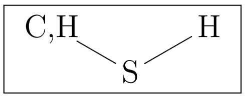

File: out/050_g_50_10300000

0.1.52 thiol_base_0

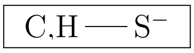

File: out/051_g_51_10300000

0.1.53 selenol

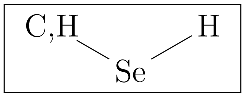

File: out/052_g_52_10300000

0.1.54 selenol_base_0

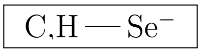

File: out/053_g_53_10300000

0.1.55 sulfide

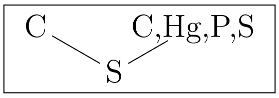

File: out/054_g_54_10300000

0.1.56 sulfide_acid_0

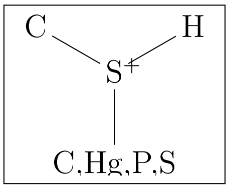

File: out/055_g_55_10300000

0.1.57 disulfide

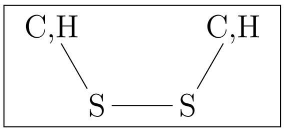

File: out/056_g_56_10300000

0.1.58 disulfide_base_0

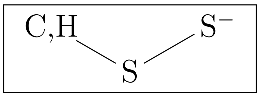

File: out/057_g_57_10300000

0.1.59 sulfanyl alkanone

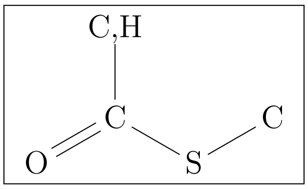

File: out/058_g_58_10300000

0.1.60 sulfanyl alkanol

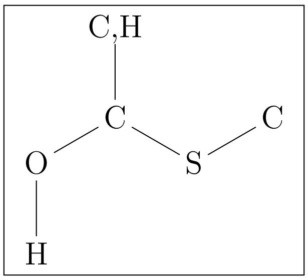

File: out/059_g_59_10300000

0.1.61 sulfanyl alkanol_base_0

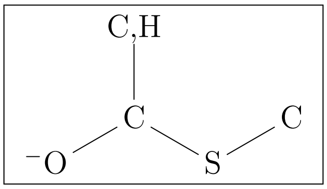

File: out/060_g_60_10300000

0.1.62 sulfoxide

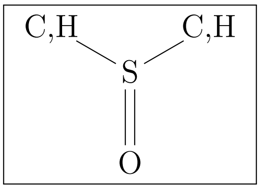

File: out/061_g_61_10300000

0.1.63 sulfonium

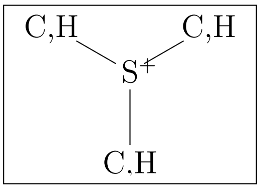

File: out/062_g_62_10300000

0.1.64 sulfurous acid_base_0

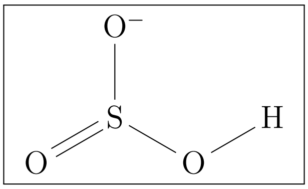

File: out/063_g_63_10300000

0.1.65 sulfurous acid_base_1

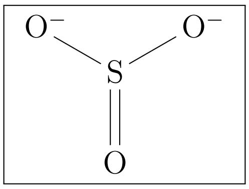

File: out/064_g_64_10300000

0.1.66 sulfuric acid_base_0

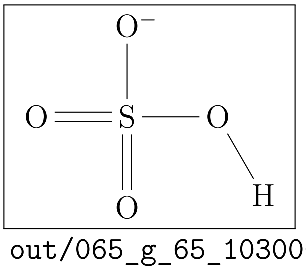

File: out/065_g_65_10300000

0.1.67 sulfuric acid_base_1

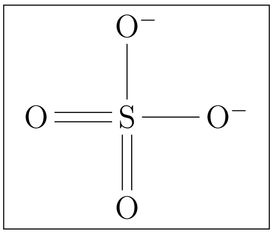

File: out/066_g_66_10300000

0.1.68 sulfonic acid_base_0

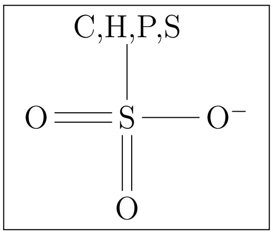

File: out/067_g_67_10300000

0.1.69 sulfonate ester

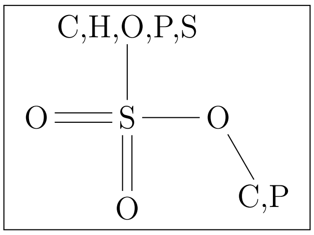

File: out/068_g_68_10300000

0.1.70 thial

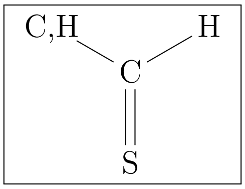

File: out/069_g_69_10300000

0.1.71 phosphine

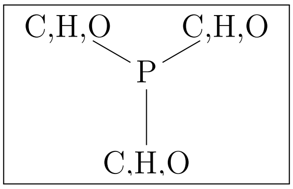

File: out/070_g_70_10300000

0.1.72 phosphoric acid_base_0

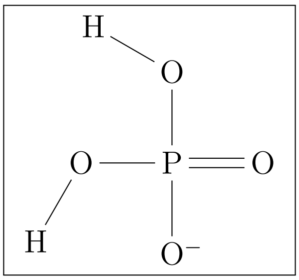

File: out/071_g_71_10300000

0.1.73 phosphoric acid_base_1

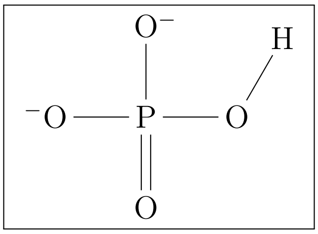

File: out/072_g_72_10300000

0.1.74 phosphoric acid_base_2

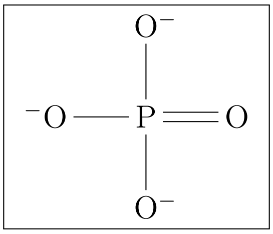

File: out/073_g_73_10300000

0.1.75 metaphosphate

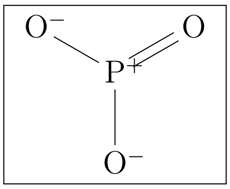

File: out/074_g_74_10300000

0.1.76 phosphoester

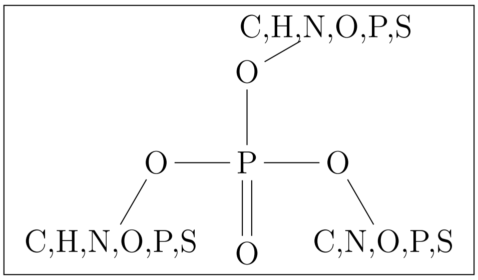

File: out/075_g_75_10300000

0.1.77 phosphoester_base_0

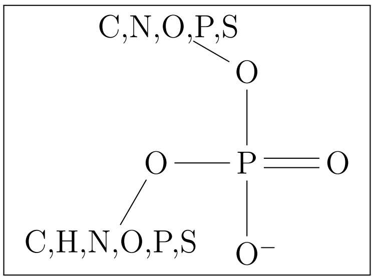

File: out/076_g_76_10300000

0.1.78 phosphoester_base_1

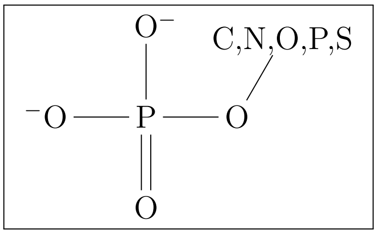

File: out/077_g_77_10300000

0.1.79 thiophosphoester_base_1

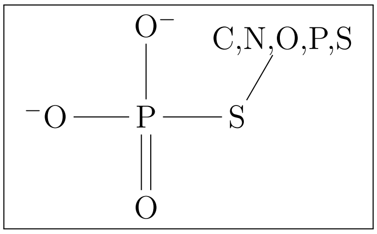

File: out/078_g_78_10300000

0.1.80 pentahydroxyphosphorane_base_1

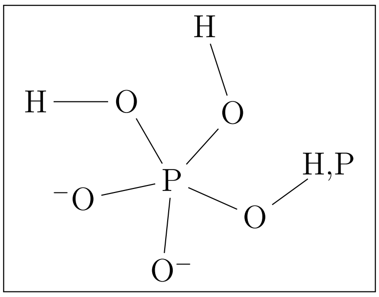

File: out/079_g_79_10300000

0.1.81 pentahydroxyphosphorane_base_2

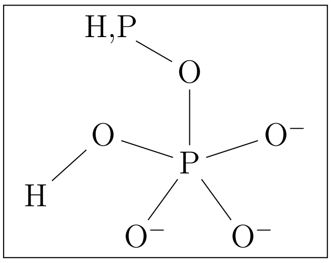

File: out/080_g_80_10300000

0.1.82 tetrahydroxyphosphorane_base_2

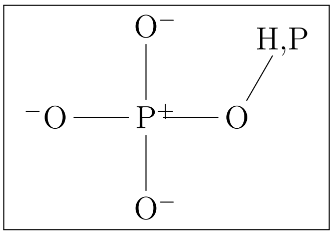

File: out/081_g_81_10300000

0.1.83 hydroxyoxophosphoniumolate_base_0

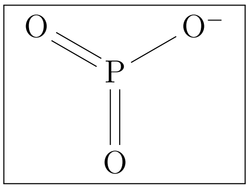

File: out/082_g_82_10300000

0.1.84 vanadate

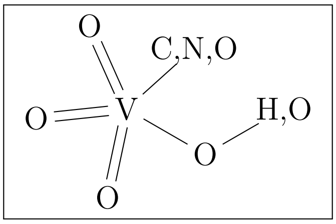

File: out/083_g_83_10300000

0.1.85 vanadate_acid_0

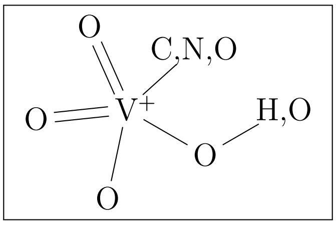

File: out/084_g_84_10300000

0.1.86 vanadate_acid_1

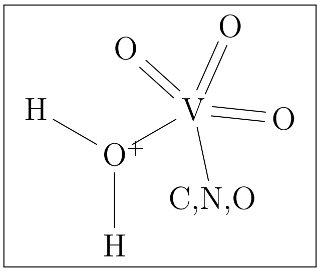

File: out/085_g_85_10300000

0.1.87 peroxovanadate

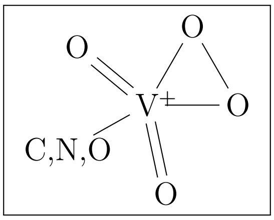

File: out/086_g_86_10300000

0.1.88 peroxovanadate_base_0

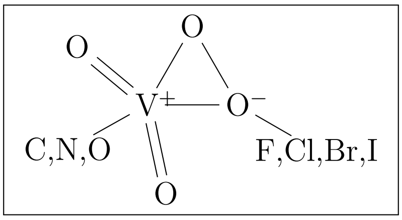

File: out/087_g_87_10300000

0.1.89 tetrahydrofuran

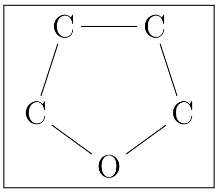

File: out/088_g_88_10300000

0.1.90 tetrahydrofuran_acid_0

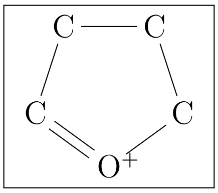

File: out/089_g_89_10300000

0.1.91 oxacyclopentene

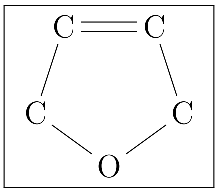

File: out/090_g_90_10300000

0.1.92 butyrolactone

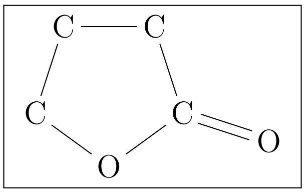

File: out/091_g_91_10300000

0.1.93 dioxolane

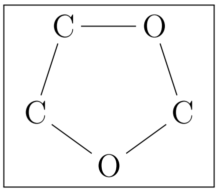

File: out/092_g_92_10300000

0.1.94 dioxolane-2-ol

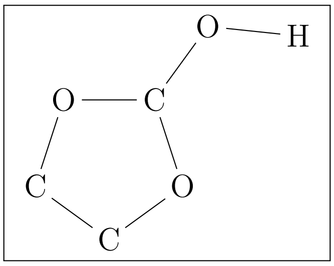

File: out/093_g_93_10300000

0.1.95 dioxolane-2-ol_acid_0

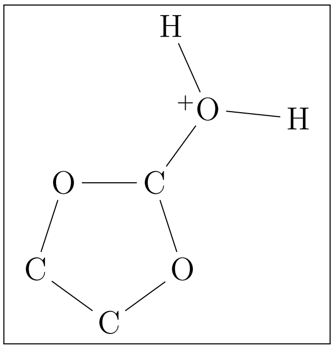

File: out/094_g_94_10300000

0.1.96 tetrahydropyran

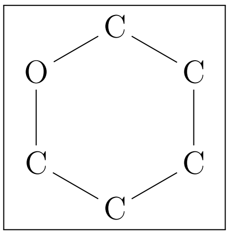

File: out/095_g_95_10300000

0.1.97 tetrahydropyran_base_0

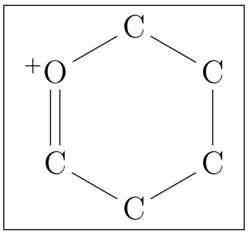

File: out/096_g_96_10300000

0.1.98 tetrahydropyran_base_1

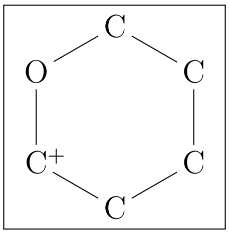

File: out/097_g_97_10300000

0.1.99 dihydropyran

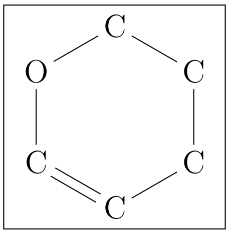

File: out/098_g_98_10300000

0.1.100 valerolactone

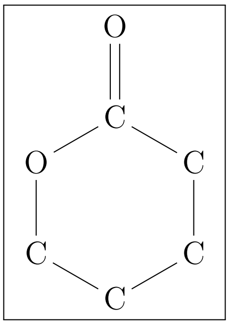

File: out/099_g_99_10300000

0.1.101 tetrahydropyran-3-one

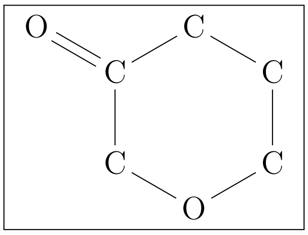

File: out/100_g_100_10300000

0.1.102 4-oxotetrahydropyran

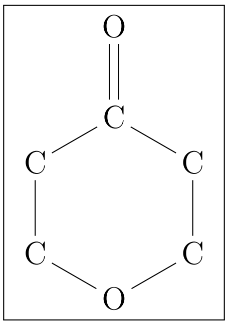

File: out/101_g_101_10300000

0.1.103 azetidinol

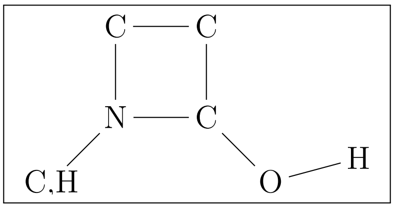

File: out/102_g_102_10300000

0.1.104 azetidinol_base_1

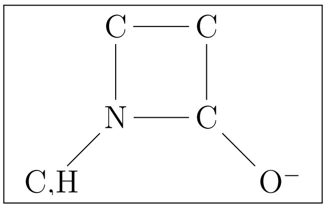

File: out/103_g_103_10300000

0.1.105 azacyclobutanone

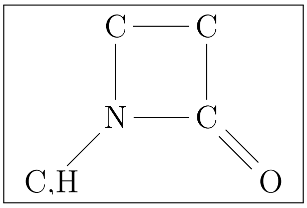

File: out/104_g_104_10300000

0.1.106 pyrrolidine

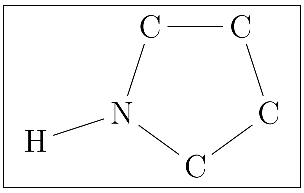

File: out/105_g_105_10300000

0.1.107 `pyrrole`

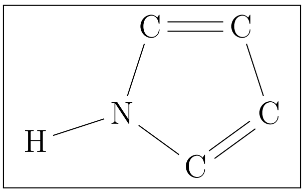

File: `out/106_g_106_10300000`

0.1.108 `imidazole`

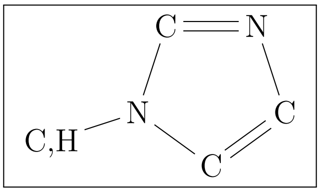

File: `out/107_g_107_10300000`

0.1.109 `imidazole_acid_0`

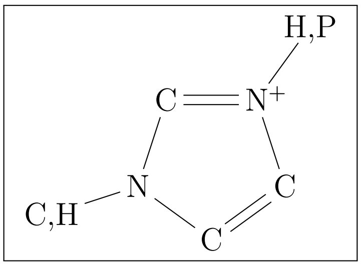

File: `out/108_g_108_10300000`

0.1.110 `imidazole_base_0`

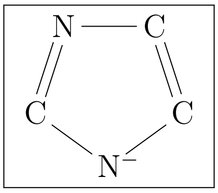

File: `out/109_g_109_10300000`

0.1.111 `monophosphoimidazole_base_1`

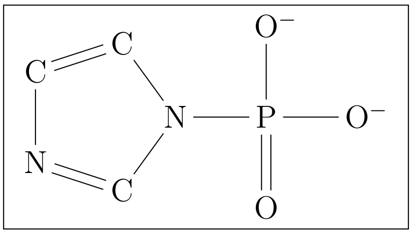

File: `out/110_g_110_10300000`

0.1.112 imidazoline

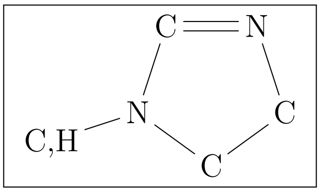

File: out/111_g_111_10300000

0.1.113 imidazoline_acid_0

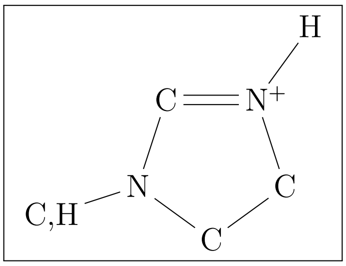

File: out/112_g_112_10300000

0.1.114 imidazoline_base_0

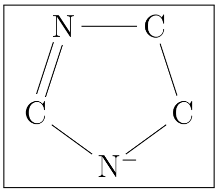

File: out/113_g_113_10300000

0.1.115 imidazol-2-ine

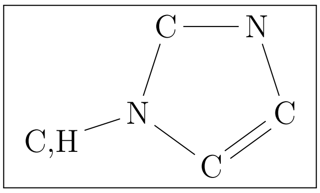

File: out/114_g_114_10300000

0.1.116 imidazol-2-ine_base_0

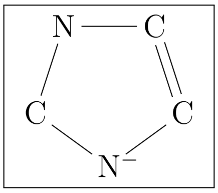

File: out/115_g_115_10300000

0.1.117 piperidine

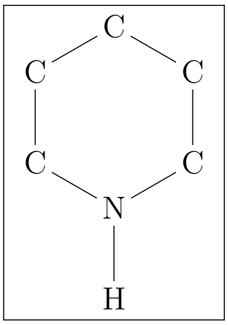

File: out/116_g_116_10300000

0.1.118 piperideine

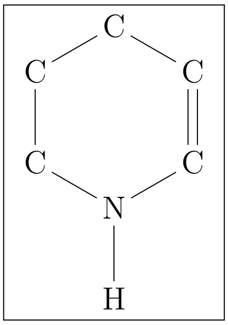

File: out/117_g_117_10300000

0.1.119 pyridine

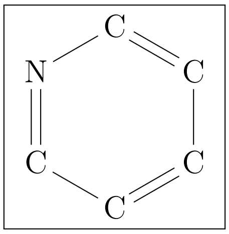

File: out/118_g_118_10300000

0.1.120 pyridine_acid_0

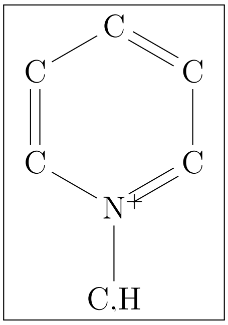

File: out/119_g_119_10300000

0.1.121 1,2-dihydropyridine_acid_0

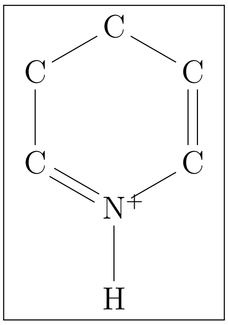

File: out/120_g_120_10300000

0.1.122 3,4-dihydropyridine

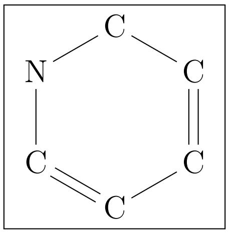

File: out/121_g_121_10300000

0.1.123 1,4-dihydropyridine

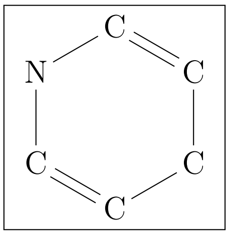

File: out/122_g_122_10300000

0.1.124 pyrimidine

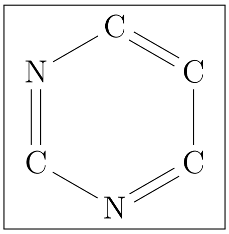

File: out/123_g_123_10300000

0.1.125 pyrimidine_acid_0

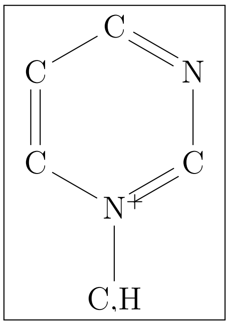

File: out/124_g_124_10300000

0.1.126 2-alkylpyrimidine

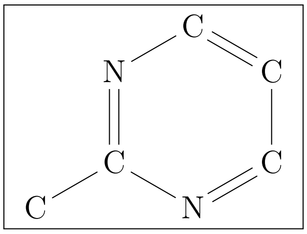

File: out/125_g_125_10300000

0.1.127 dihydropyrimidine

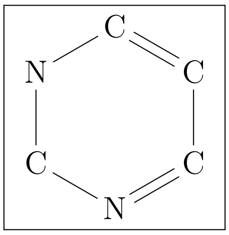

File: out/126_g_126_10300000

0.1.128 dihydropyrimidine_acid_0

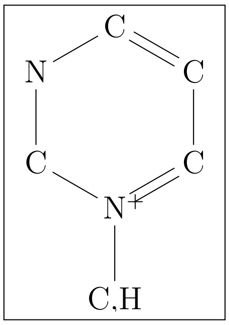

File: out/127_g_127_10300000

0.1.129 3,4,5,6-tetrahydropyrimidine

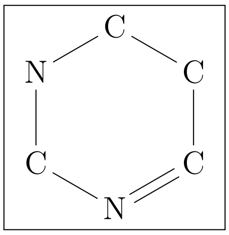

File: out/128_g_128_10300000

0.1.130 3,4,5,6-tetrahydropyrimidine_base_0

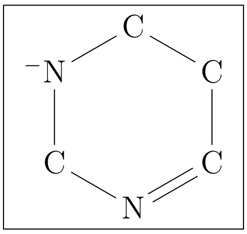

File: out/129_g_129_10300000

0.1.131 4-pyrimidinone

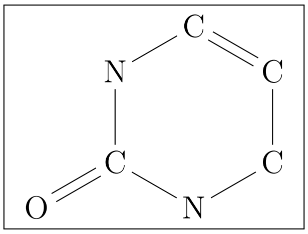

File: out/130_g_130_10300000

0.1.132 hydroxy-4-pyrimidinone

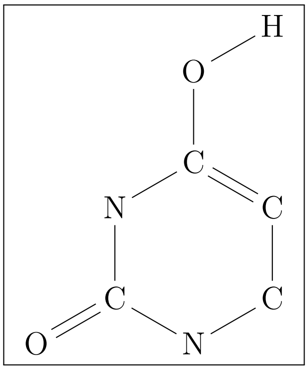

File: out/131_g_131_10300000

0.1.133 hydroxy-4-pyrimidinone_base_0

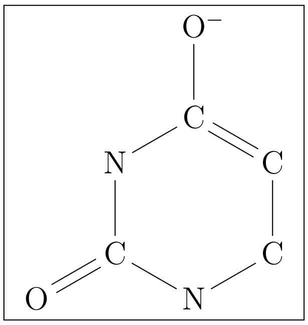

File: out/132_g_132_10300000

0.1.134 uracil

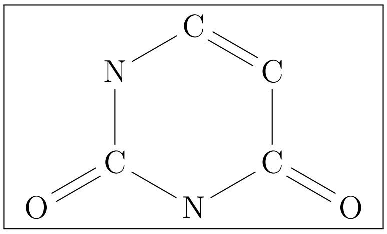

File: out/133_g_133_10300000

0.1.135 uracil_base_0

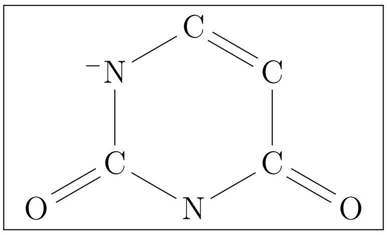

File: out/134_g_134_10300000

0.1.136 pyrazine

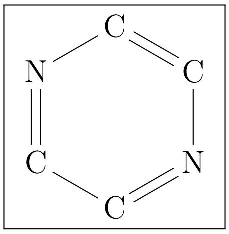

File: out/135_g_135_10300000

0.1.137 1,2-dihydropyrazine

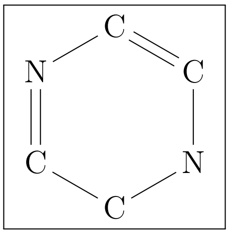

File: out/136_g_136_10300000

0.1.138 1,2-dihydropyrazine_acid_0

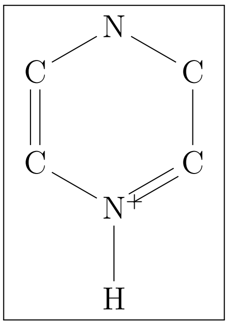

File: out/137_g_137_10300000

0.1.139 2,3-dihydropyrazine

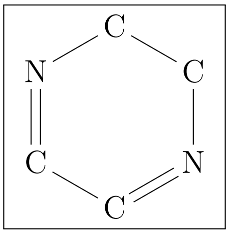

File: out/138_g_138_10300000

0.1.140 2,3-dihydropyrazine_acid_0

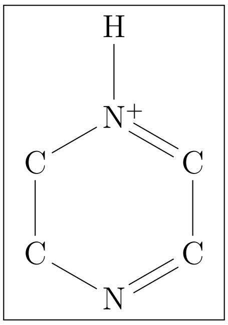

File: out/139_g_139_10300000

0.1.141 1,4-dihydropyrazine

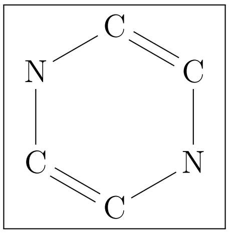

File: out/140_g_140_10300000

0.1.142 tetrahydropyrazine

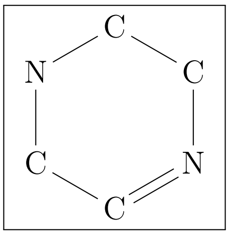

File: out/141_g_141_10300000

0.1.143 thiazole_acid_0

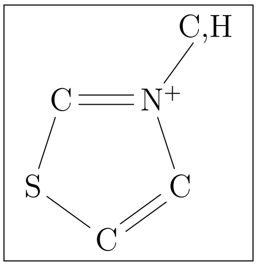

File: out/142_g_142_10300000

0.1.144 thiazolidine

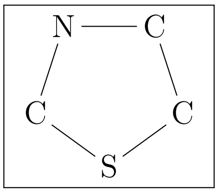

File: out/143_g_143_10300000

0.1.145 thiazolidine_base_0

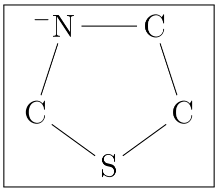

File: out/144_g_144_10300000

0.1.146 2,4-cyclohexadienone

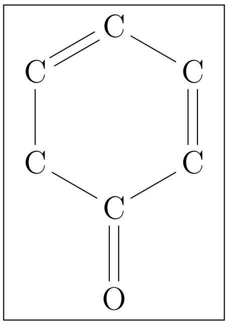

File: out/145_g_145_10300000

0.1.147 2,5-cyclohexadienone

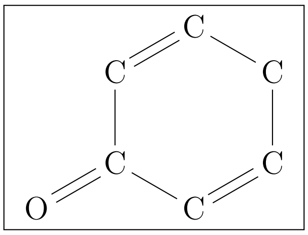

File: out/146_g_146_10300000

0.1.148 2,5-cyclohexadienone_acid_0

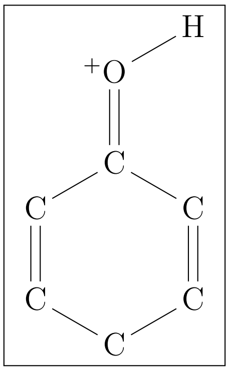

File: out/147_g_147_10300000

0.1.149 halobenzene

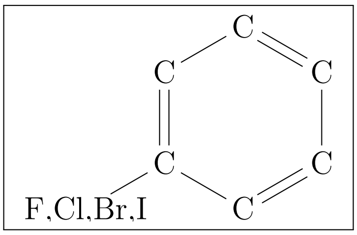

File: out/148_g_148_10300000

0.1.150 toluene

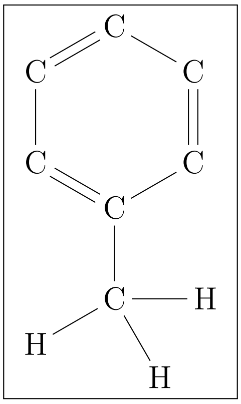

File: out/149_g_149_10300000

0.1.151 ortho-xylene

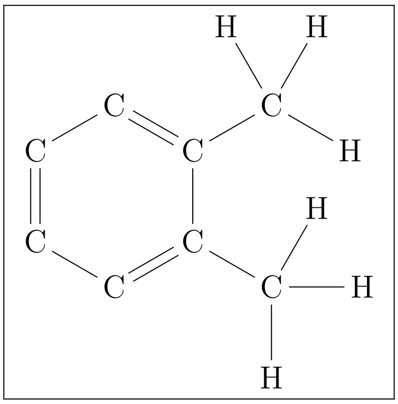

File: out/150_g_150_10300000

0.1.152 phenol

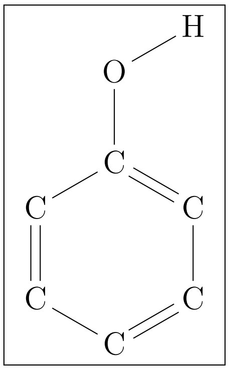

File: out/151_g_151_10300000

0.1.153 phenol_base_0

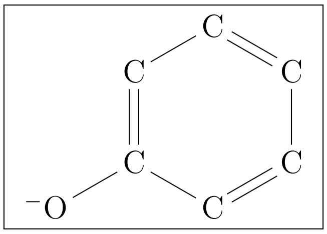

File: out/152_g_152_10300000

0.1.154 benzaldehyde

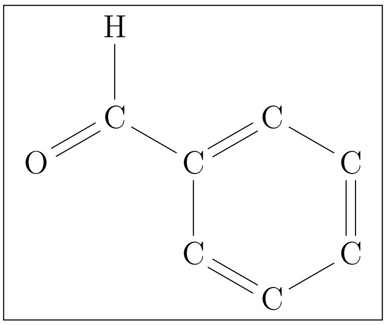

File: out/153_g_153_10300000

0.1.155 benzyl alcohol

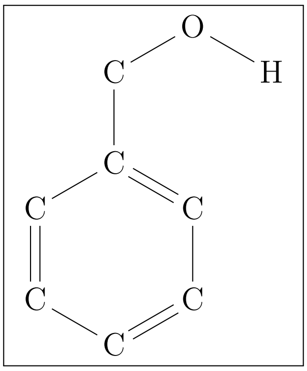

File: out/154_g_154_10300000

0.1.156 benzyl alcohol_base_0

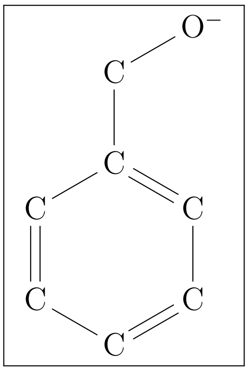

File: out/155_g_155_10300000

0.1.157 anisole

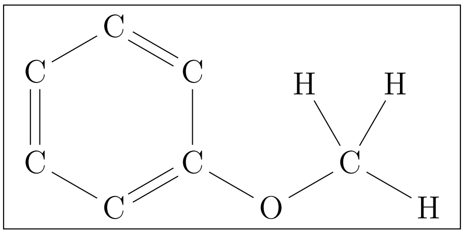

File: out/156_g_156_10300000
